# Status, Stress and Performance in Track and Field Athletes during the European Games in Baku (Azerbaijan)

**DOI:** 10.1038/s41598-017-06461-z

**Published:** 2017-07-20

**Authors:** Benjamin Siart, Alfred Nimmerichter, Claudia Vidotto, Bernard Wallner

**Affiliations:** 1 Department of Anthropology, University of Vienna, Althanstrasse 14, 1090 Vienna, Austria; 2 Department of Behavioural Biology, University of Vienna, Althanstrasse 14, 1090 Vienna, Austria; 3 Faculty of Training and Sports Sciences, University of Applied Sciences Wiener Neustadt, Johannes Gutenbergstrasse 3, 2700 Wiener Neustadt, Austria; 4 Study Lab G.m.b.H., Davidgasse 87-89, 1100 Vienna, Austria

## Abstract

This study analyzes the relationship between salivary cortisol and testosterone levels and performance in track and field athletes. In addition, we analyzed the influence of status among athletes (measured based on previous athletic achievement) on hormone levels. Nineteen members of the Austrian track and field team (eleven males, eight females, 25.9 ± 3.9 years of age, 74.9 ± 20.1 kg, and 179.3 ± 10 cm) participated in this study. Data was collected during the European Games in Baku. Athletes delivered saliva samples at various time-points including morning samples and samples directly before and after the competition. Scoring points of the International Association of Athletics Federation were used as an individual measure of relative performance. We found that performance was negatively correlated with rise in testosterone concentrations in the last 24 h prior to the competition. A similar trend was found for cortisol levels, but only when the three least competitive athletes were removed from analysis. Pre-competition cortisol levels were significantly increased compared to measurements 24 h earlier. No effect of status on cortisol or testosterone increase in the same timeframe was found. We conclude that the tournament represented a stressor and that excessive endocrine response was associated with reduced performance.

## Introduction

Social status affects behavior and hormone levels in humans and other nonhuman species^1, 2^. Magee and Galinsky^3^ define status as “the extent to which an individual or group is respected or admired by others”. Humans are typically part of multiple hierarchy systems and tend to attach the highest value to the system in which they hold the highest social status^2^. In competitive athletes, this most valued hierarchy is their peer environment, making status among other athletes very important to them. In such a setting, status depends greatly on athletic performance, and sports-competitions can be seen as formalized contests for status^4^. It has been argued that the pressure to perform well in a competition poses a social-evaluative threat, that is, a threat to a person’s social status^5^. Such a challenge can be stronger than other perceived stressors. For instance, competition among a cohort of ballroom dancers causes physiological stress responses, as measured based on salivary cortisol, that are greater than the Trier social stress test, a well-established laboratory stressor^5^. Competitive environments may represent severe stressors because the outcome of a competition can influence an individual’s self-esteem and self-identity. Furthermore, negative evaluation during and after competitions leads to a loss of social status in a cherished domain^5^. Sports competitions are therefore an ideal scenario to study the interaction between challenges and social statusN in a cherished domain.

Testosterone has been widely analyzed in studies on social status and dominance in humans and nonhuman primates^6, 7^. In this regard it has been argued that testosterone influences aggressive and dominant behavior^8^ and increases the motivation to seek status^9^. It has also been hypothesized that testosterone levels rise in an anticipatory response to an upcoming social challenge such as a competition^10^ and that the outcome of the challenge modulates testosterone levels^8^. A study on male and female soccer players reported ratings of playing abilities, used as a measure of status among teammates, to be positively correlated with game related testosterone levels in both sexes^4^. Regarding the outcome of the challenge, several studies showed that victory led to an increase in testosterone, whereas losing led to an increase in cortisol and a decrease in testosterone^11, 12^. Other studies found the effects on these hormones to be reversed or found no effect^7, 12^. Research on competition outcomes is mainly conducted with team sports or sports with dyadic interactions such as tennis or boxing. In these sports an individual’s performance and the outcome of the competition depend greatly on the actions of the opposition and the individual’s team mates^11–14^. Note here that the performance of an individual is not equal to the outcome of the competition. For example in team sports an individual may perform poorly but still win because others in the team performed well. Similarly, a tennis player may play at the peak of his or her ability and still lose a match if the opposition plays even better. A study on male and female badminton players showed that testosterone levels rose after a win and declined after a loss in both sexes. In the same study, cortisol levels were elevated after a loss and reduced after winning a match^11^. In contrast, a recent study on female soccer players did not find the same relationship between match outcome and changes in cortisol or testosterone levels^15^. To summarize, testosterone is assumed to increase after a positive outcome such as winning a challenge. No consensus, however, has been reached because a number of studies document contrary results^16–18^. Divergent results on hormone secretion rates can have several potential explanations. For example, in team sports the individuals’ contribution during the competition seems to be a better predictor of hormone secretion rates than the outcome of a competition^16^. Additional factors influencing a person’s psychological and physiological reaction to the outcome of a competition are: the importance of the competition (e.g., friendly games vs. world championships), the person’s involvement, perceived control of the outcome, and the opponents’ status, i.e. whether the person believes the opponent to be a better athlete^19^. Individual sports, where performance can be measured in a standardized way, could help to avoid many of these influencing factors. In sports such as swimming, weightlifting or track and field athletics, performance is measured in meters, seconds or kilos and opponents and teammates do not have as much of an influence on performance as in other sports.

Competitions are not only useful as models for challenges, they also represent profound physiological and psychological stressors. In general, during environmental or psycho-socially related challenges, the hypothalamic-pituitary adrenal-axis (HPA) is activated, increasing cortisol secretion rates from the adrenal gland^20^. Cortisol is a well-known steroid stress marker and well investigated in sports or exercise-related studies^21^.

If a person is challenged by a stressor, cortisol is released to prepare the body to cope in a behavioral and physiological manner. Differences in how an athlete copes with such a stressor might be reflected in their performance during a competition. More specifically, a higher activation of the stress system leads to increased arousal and attention and suppresses pain. Respiration and cardiac output are accelerated, catabolism is increased, and blood flow is redirected to provide as much energy to the brain, heart and muscles as possible^20^. Stress therefore potentially increases athletic performance. Conversely, extreme levels of arousal are thought to be detrimental to the athlete’s ability to cope adequately with competitive requirements^22^. It is often assumed that increased stress or arousal initially increases performance in a task, but that any additional increase in arousal beyond that point will negatively impact performance. This leads to the assumption that an optimal level of arousal or stress exists for any given task^23, 24^. The notion that the relationship between physiological stress and performance follows a gerneralisable pattern, however, is much disputed^25^.

For instance, the impact of cortisol, as a measure of stress, on individual athletic performance is poorly studied and the few reported results are ambiguous. In weightlifters, positive correlations between cortisol and performance were reported^26, 27^. Cortisol concentrations were found to be positively correlated to speed, power, and strength in professional rugby union players^28^. In contrast, another study on rugby players found values to be negatively correlated with the coaches’ evaluation of player performance^29^. A recent study reported cortisol levels to be negatively correlated with tennis serve performance^30^. An exploratory case study found cortisol to be negatively correlated with performance in a tennis match: this was valid only in some performance parameters such as return performance and unforced errors, but not in others such as serving performance. Intriguingly, cortisol levels in the same study were positively correlated with the number of winners (hits that are a direct point) that the players produced^31^. In golfers, cortisol was negatively correlated with performance in a golf competition^32^. The inconsistency in results may be explained partly by differences in study design. For example, the aforementioned study on tennis serve performance used a laboratory stressor to increase cortisol^30^, whereas the explorative case study on cortisol during a tennis match sampled a single match very thoroughly^31^. One rugby study performed scheduled tests^28^, whereas the other collected data following different motivational pre-game treatments before official rugby union matches^29^. The studies on weightlifting were performed during official and simulated competitions^26, 27^, the study on golf during a collegiate competition^32^. Another possible explanation for the variability of results is that the effect of increased cortisol levels varies between sports because the requirements of the sports differ. Nonethelss, little sports-related data is available compared to performance in other fields. For example, physiological stress affects decision-making performance, but the size and direction of the effect depends on the specific task and situation^33^. Perceptual motor performance deteriorates with stress but is more resilient to stress than higher-order cognitive processes such as memory. Furthermore, gross motor skills may be more resilient than fine motor skills^25^. This conclusion, however, was drawn from a review of studies utilizing a variety of stressors including fatigue, thermal stress, noise, workload, threat of electric shock, anxiety and fear, time pressure, and military combat^25^. The results may therefore not be fully applicable to correlations between cortisol and performance.

In this study we investigated the relationship between a competition and its outcome on testosterone and cortisol levels in athletes. We also examined the effect of status on changes in hormone levels in anticipation of the competition. For this purpose, we conducted a study on track and field athletes during the first European Games in Baku (Aze) an international multi-sport event in the Olympic tradition for athletes representing the National Olympic Committees of Europe.

Track and field athletes were chosen because (a) their performance is measured objectively in meters or seconds, (b) a method to compare results between disciplines has been validated for decades in the form of the International Association of Athletics Federation scoring table^34^ and (c) an opponent’s influence on individual performance is reduced and less direct compared with other sports such as tennis, boxing or team sports. The study examined (i) correlations between cortisol and testosterone secretion levels and athletic performance, (ii) whether an individual’s status among track and field athletes, defined as the athlete’s best performance of the season before the tournament, affects hormone-levels and (iii) whether the athletic performance in the tournament affects salivary levels of testosterone and cortisol after the event. No comparable study has been conducted at a competition of this magnitude.

## Methods

### Subjects

Nineteen members (eleven males 26.3 ± 3.9 years of age, 84.8 ± 20.5 kg, 184.6 ± 8.9 cm and eight females 25.5 ± 4.1 years of age, 61.3 ± 8.3 kg, 172 ± 6.3 cm) of the Austrian track and field team participated in this study. All participants were athletes starting in single events: jumps (N = 4), long distance (N = 3), middle distance (N = 2), sprint (N = 5), throws (N = 5). Participants had to be above eighteen years of age in order to be included in the study. Athlete’s health was determined by the Austrian Olympic Committee (ÖOC) prior to selection into the team. Before entering the study, participants were informed of all procedures in the study and gave written informed consent to participate. One athlete encountered faulty equipment during the competition and was therefore removed from statistical analysis that includes performance at the EG. Athletes who provided no saliva samples at the time points used in the analyses, did not have a season’s best performance in their discipline, or did not start in a single event, were not included in the study.

The track and field competition at the European Games was held according to the rules and as a part of the European Athletics Team Championships in Athletics. This had the advantage of a relatively large number of athletes competing at an important occasion on two consecutive afternoons, under stable weather conditions. In this competition mode one male and one female athlete per country starts in each discipline to score points for his or her nation. Since not every nation has a professional athlete for every discipline, the level of ambition and competitiveness varies between athletes. In the cohort of this study three athletes reported not to be fully active, competitive athletes. These athletes also had the lowest season’s best performance in the cohort (see Supplementary Figs 1 and 2). Therefore, these athletes are highlighted in the plots and analyses.

The study was conducted in accordance with the declaration of Helsinki and was submitted to the ethics committee of the City of Vienna (Unit 15, Health and Care, reference number: EK_15–121-VK_NZ). The ethics-committee cited Article15a paragraph 3a of the Viennese healthcare law, stating that for this kind of study no further processing by an ethics committee was needed. All research protocols were reviewed and approved by the commission of the Austrian athletics federation (OELV, reference number: 6495). The decision was that all ethical and research guidelines of the federation were fulfilled. All participating athletes gave written informed consent for all procedures in the study. Athletes were given questionnaires and supplied information about age, gender, weight, height, previous athletic career including SB, years of participation and time invested in the sport.

### Saliva sampling and analysis

**Figure 1:**
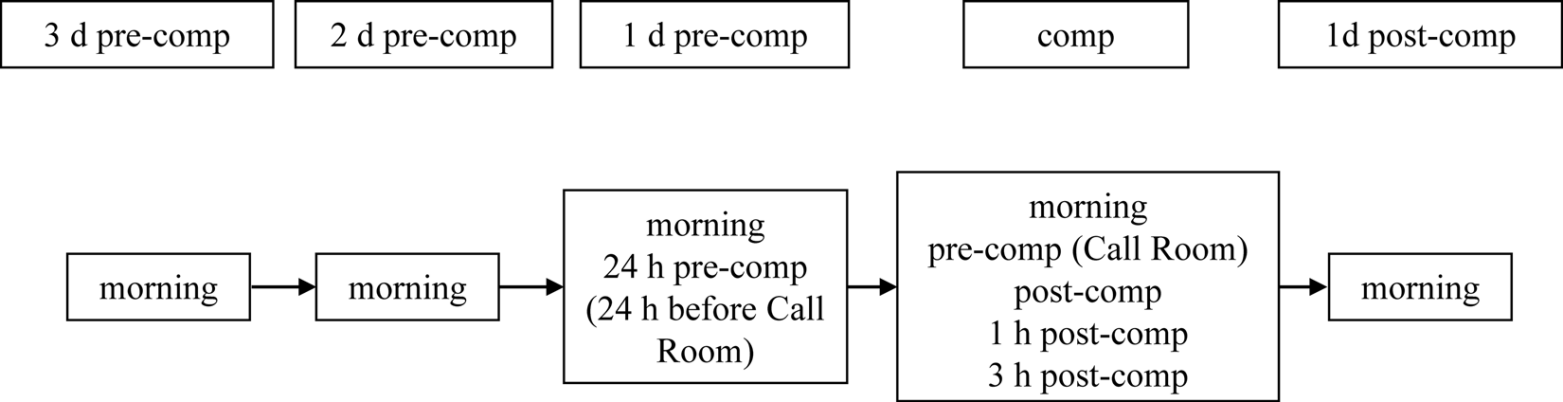
Saliva sample schedule. Upper boxes represent day of sampling, lower boxes state the sampling times of the respective day. Upper boxes: 3 d, 2 d and 1 d pre-comp = three, two and one day before the day of the competition, respectively, comp = day of the competition, 1 d post-comp = first day after the competition. Lower boxes: morning = morning of the sampled day directly after waking up, 24 h pre-comp = 24 h before entering the Call Room, pre-comp = before entering the Call Room, post-comp = immediately after competition, post-comp 1 h = 1 h after competition, post-comp 3 h = 3 h after competition.

Saliva samples for hormonal analysis were collected using SaliCaps (IBL International, Hamburg, Germany). Saliva samples were used for three main reasons. First, the Olympic Committee has a strict no needle policy that prohibits the use of syringes at competition and training sites. Second, venipuncture can cause hematoma which might reduce limb function and cause pain. Third, salivary cortisol is a better measurement of dynamic HPA activity than serum cortisol^35^. The second point is especially important to athletes before a major competition, and venipuncture would reduce compliance in participants. Athletes were instructed to supply ten saliva samples at several time points during a period of 5 days including the competition day. Athletes arrived in Baku between two and six days before the competition. All athletes travelled from Austria, thus crossing one time zone on their way to Azerbaijan. The sample collection schedule was as follows: the last three days before the athlete’s competition, the day of the competition and the day after the competition. Morning samples were collected on each of the sampled days immediately after the athlete woke up. Pre-competition samples (pre-comp) were taken when the athletes entered the Call-Room (The Call Room is the last stop before an athlete enters the stadium and starts the last preparations for the competition. Depending on the discipline, the Call Room is entered 25–65 min before the start of the competition). Pre-comp samples were compared to samples taken at the same time on the day before the competition (24 h pre-comp; 24 h before entering the Call Room). Additional samples were collected immediately after, 1 h after and 3 h after (1 h and 3 h post-comp) the competition (Fig. 1).

Competitions began at 16:00 h on both days and the last individual competition started at 20:30 on the first day and at 20:05 h on the second day. To study the effect on competition on hormone levels, samples were taken exactly 24 h before the competition (24 h pre-comp) and compared with the sample taken before the competition. Thereby the control sample was taken at the same time of day as the competition sample. This is an important measure to counteract the effect of daily hormone fluctuations and different starting times of the individual competitions.

The saliva samples were kept cool with dry ice on site and during shipment to the lab, where they were stored at −20 °C. Cortisol levels were analyzed by a medical laboratory using the Access Cortisol Assay on the Access Immunoassay-System platform (Beckman Coulter, Brea, California). This fully automated CE marked assay is used regularly for human medical applications. Measurements were performed according to manufacturer instructions. In accordance with the instructions the samples were run in singlets. All samples were measured in a single run; the intra-assay coefficient of variation was 4.4%. Testosterone was analyzed using IBL ELISA Testosterone kits (International, Hamburg, Germany). Testosterone assays were performed in duplicates and according to the manufacturer’s manual. For testosterone, the intra-assay coefficient of variation was 6.35% and the inter-assay variation was 3.05%. Salvia samples were used for cortisol analyses, yielding 10 sample points. The same samples were used to analyze testosterone levels in a subsequent analysis. The remaining amount of saliva available for the testosterone assay did not allow for analyzing all data points. The sample points for the testosterone assay were: 24 h pre-comp, morning of the competition, pre-comp, morning post-comp.

### Measurements of performance and status

Seasons best performance was converted into scoring points using the IAAF scoring table^34^. When athletes reported indoor results as their season’s best, the appropriate table was used to convert the performance into scoring points. To compare how well each individual athlete performed during the games, each athlete’s European Games Performance in scoring points (*EGP*) was transformed into a delta-value using the athlete’s self-reported season’s best performance in scoring points (*SBP*) before the games. Equation 1. Was used to derive an athlete’s relative performance (Δ*P*).

1 $${\rm{\Delta }}P=\frac{EGP}{EGP+SBP}$$

Δ*P* = athlete’s relative performance at the European Games; *EGP* = performance at the European Games in IAAF scoring points; *SBP* = season’s best performance before the European Games in IAAF scoring points.

The resulting Δ*P* is a dimensionless number between 0 and 1. The resulting number is greater the better the performance at the competition was relative to the best performance of the season before the European Games. A value of 0.5 represents a EGP equal to the SBP. Numbers above 0.5 represent performances that were better than the season’s best performance, whereas values below 0.5 represent performances worse than the athlete’s season’s best. The athlete’s *SBP* was used as a measure of status among athletes, assuming that better performance translates to status among athletes.

### Data analysis

Descriptive results are presented as mean ± standard deviation. Non-parametric tests were used for correlation analysis (Spearman’s rank correlation coefficient) and to test for significant differences between sample points (Mann-Whitney –*U* test or Wilcoxon matched pairs signed-rank test). Spearman’s rank correlation coefficient was chosen because it is more robust towards outliers than parametric analyses and is not based on the assumption of normality. All statistical analyses were conducted in *R* with α set at 0.05. Due to the relatively small sample size, significant correlations were bootstrapped to further test the validity of the results. The bootstrapped 95% confidence interval (95% CI) is displayed in addition to the *r*_*s*_ value of the bootstrapped correlation. Bootstrapping was performed utilizing the *boot* package in *R* with 10,000 repeats. The power of significant and marginally significant results was estimated using the *pwr*.*r*.*test* function in the *pwr* package in *R* with alpha set to 0.05. Graphical illustrations were created using the packages *ggplot2*, *cowplot*, *beewswarm* and *extrafont* in *R*.

Delta values were used for all statistical analyses of hormones. This was done in order to reduce inter-personal and inter-sexual variation. Equation 2 was used to derive delta values in hormones.

2 $${\rm{\Delta }}H=\frac{H\,later}{H\,later+H\,earlier}$$

Equation 2 Delta values in hormones. Δ*H* = change in hormone level from earlier to later time point. *Hlater* = hormone level at later time point; *Hearlier* = hormone level at earlier time point.

Changes in cortisol (Δ*C)* and testosterone (Δ*T*) levels were calculated as the changes from the 24 h pre-sample to the pre-comp sample point (see section ‘Saliva Sampling and Analysis’). Changes in morning hormone levels from the morning of the competition to the day after the competition were calculated for cortisol (Δ*C*_*Morning*_) and testosterone (Δ*T*_*Morning*_). Thus, all Δ*H* values reflect the change of hormone levels in the course of 24 h, thereby representing normalized hormone levels for each individual. Δ*H* is a dimensionless number between 0 and 1, with values above 0.5 representing an increase in hormone levels, numbers below 0.5 a decrease in hormone levels between the two measured time points.

## Results

### Performance in the competition

The average Δ*P* was 0.495 (±0.01, N = 18). Performance in the competition was therefore slightly lower than season’s best performance. Six athletes achieved results that were better than their previous season’s best (Δ*P* > 0.5) whilst the other athletes in the cohort performed worse.

### Hormonal measures

Testosterone and cortisol concentrations were analyzed from the same sample material, but due to the small volume of some samples, the number of values differs for some sample points (see Supplementary Tables 1 and 2).

**Figure 2:**
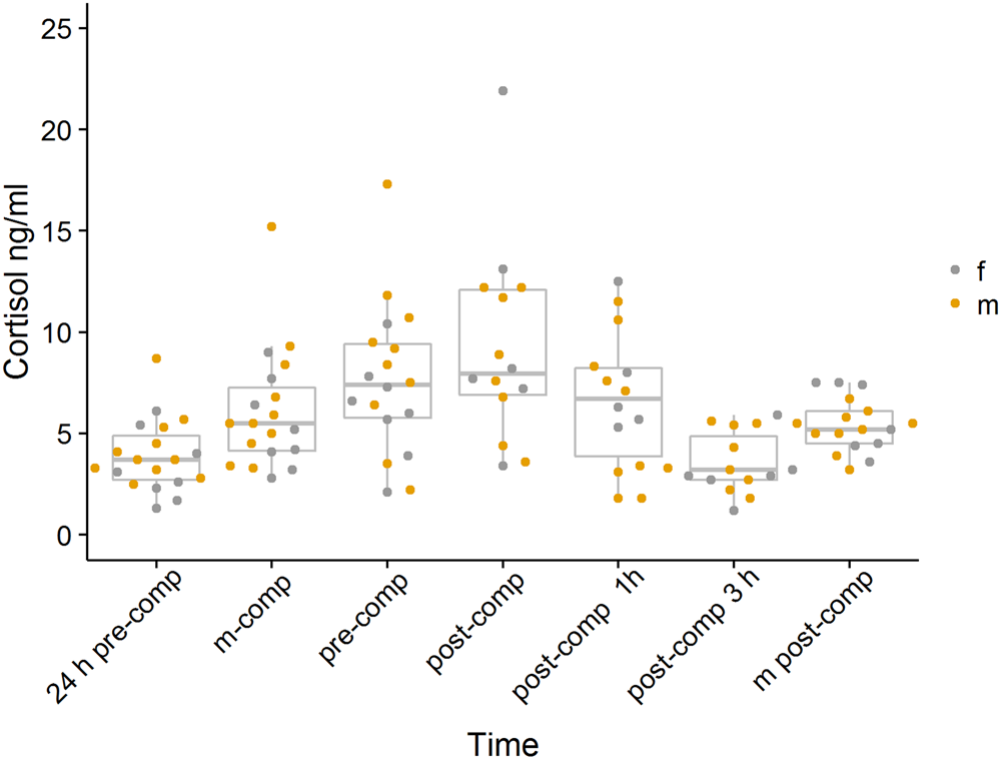
Time course of salivary C levels. 24 h pre-comp = 24 h before the Call Room. M-comp = morning of competition; pre-comp = before entering the Call Room; Post-comp = immediately after competition; post-comp 1 h = 1 h after competition; post-comp 3 h = 3 h after competition; m post-comp = morning after competition. Dots represent individual values, colour indicates sex.

Saliva cortisol levels increased significantly from 24 h before the competition (3.89 ± 1.77 ng/ml) to immediately before the competition (7.57 ± 3.72 ng/ml, Wilcoxon matched pairs signed-rank test, *P* = 0.005; *V* = 20, *r* = −0.67, *N* = 18). Cortisol levels in the time course of the experiment are depicted in Fig. 2. Testosterone levels did not change significantly between those two time points (24 h pre-comp: 0.84 ± 0.67 ng/ml; pre-comp: 1.33 ± 1.46 ng/ml, Wilcoxon matched pairs signed-rank test, *P* = 0.32, *V* = 36, *r* = −0.27, *N* = 14). Morning cortisol levels did not differ greatly between the sampled days (see Supplementary Table 1).

There was no significant difference in Δ*H* values between the sexes. (Mann-Whitney –*U* test, Δ*C*: *P* = *0*.*82*; *W* = *43*, *r* = −0.05, *N*_male_ = 10, *N*_female_ = 8; Δ*T*: *P* = 0.42, *W* = *16*, *r* − 0.21, *N*_male_ = 9, *N*_female_ = 5; Δ*T*_*Morning*_: *P* = 0.42, *W* = 29, *r* = −*0*.*21*, *N*_male_ = 9, *N*_female_ = 5).

### Performance and changes in hormone levels before the competition

**Figure 3:**
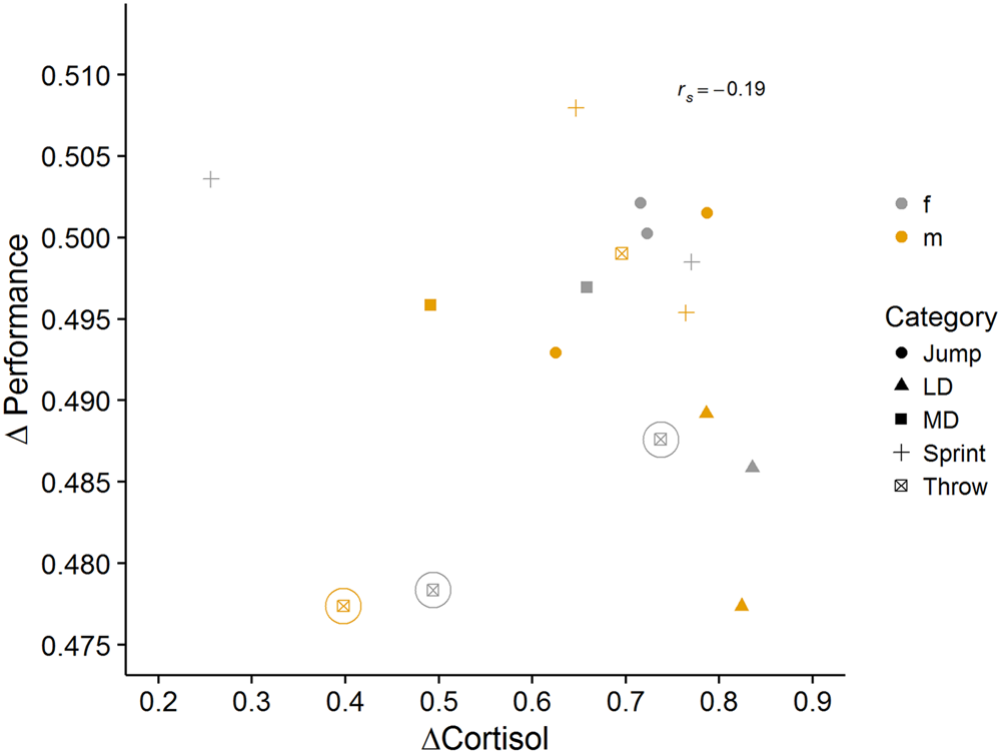
Correlation of cortisol change prior to competition, ΔC (X-axis) and performance; ΔP (Y-axis). Each symbol represents an individual athlete; color indicates sex of the athlete, shape indicates the category the athlete started in, LD = long distance running, MD = middle distance running, Jump = jumps, Sprint = sprints, Throw = throws. Athletes marked with a circle were less involved in competitive athletics than the other athletes.

There was no significant correlation between Δ*P* and Δ*C* values (*P* = 0.46, *r*_*s*_ = −0.19, *N* = 17). However, when three athletes who did not categorize themselves as fully active competitive athletes, and who had the lowest SBP in the cohort, were removed from the analysis, a negative trend between Δ*P* and Δ*C* emerged (*P* = 0.08, *r*_*s*_ = −0.49, *N* = 14 bootstrapped confidence intervals 95% CI [−0.87; 0.17], power = 0.45; Fig. 3). Here, athletes with a greater increase in cortisol in the last 24 h before the competition tended to perform worse in the competition.

**Figure 4:**
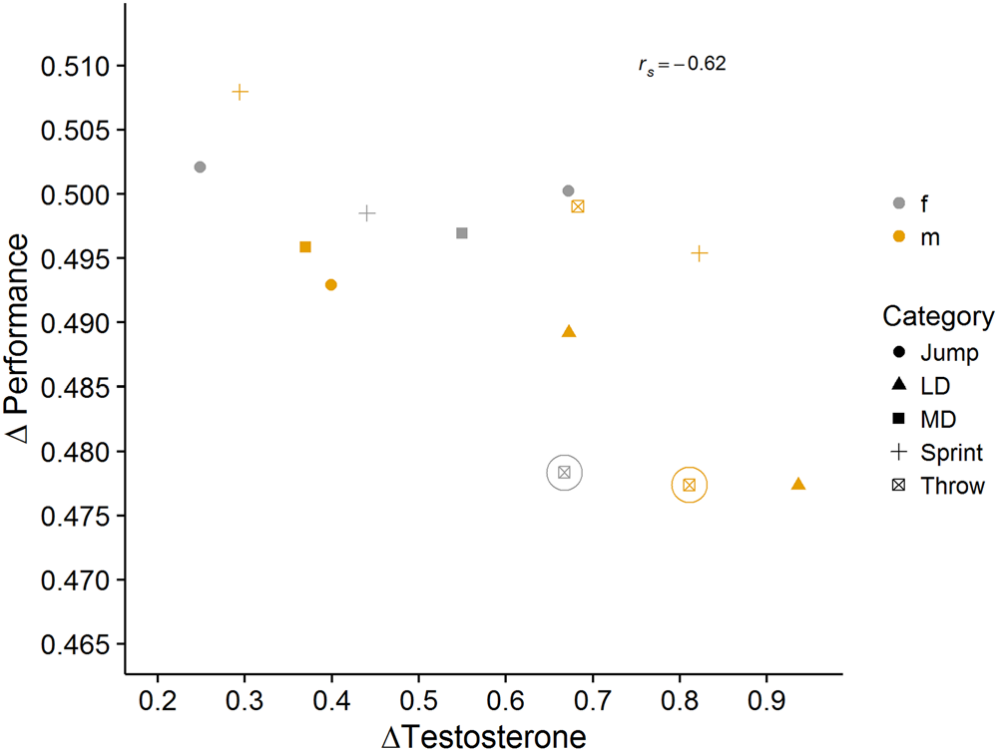
Correlation of testosterone change prior to competition, ΔT (X-axis) and performance; ΔP (Y-axis). Each symbol represents an individual athlete; color indicates sex of the athlete, shape indicates the category the athlete started in, LD = long distance running, MD = middle distance running, Jump = jumps, Sprint = sprints, Throw = throws. Athletes marked with a circle were less involved in competitive athletics than the other athletes.

There was a significant, negative correlation between Δ*T* and Δ*P* (*P* = 0.027; *r*_*s*_ = −0.62, *N* = 13, bootstrapped confidence intervals 95% CI [−0.91; −0.04], power = 0.66; Fig. 4). When only active, competitive athletes were included in the analysis the correlation between Δ*T* and Δ*P* was marginally significant (*P* = 0.066; *r*_*s*_ = −0.58, *N* = 11, bootstrapped confidence intervals 95% CI −0.91; 0.16], power = 0.49; Fig. 4). Here, a greater increase in testosterone in the last 24 h of the competition is correlated with a decrease in performance.

### Hormonal response and athletic status

The individual athlete’s *SBP* (our measure of status in athletes) was not significantly correlated with Δ*C* (*P* = 0.48, *r*_*s*_ = 0.18, *N* = 18) or Δ*T* (*P* = 0.76, *r*_*s*_ = −0.09, *N* = 14; see Supplementary Figs 1 and 2). There was also no significant correlation between change in morning testosterone levels (Δ*T*_*Morning*_) and performance in the competition (Δ*P*) (*P* = 0.63; *r*_*s*_ = −0.14, *N* = 14, Supplementary Fig. 3).

## Discussion

In this study, we detected a negative association between athletic performance and increases in testosterone levels from 24 h before the competition to immediately before the competition. A similar trend was found for cortisol levels, but only when those athletes who did not self-identify as fully active, competitive athletes (and who also had the lowest SBP in the cohort) were removed from the analysis. This suggests that an increase in these two hormones prior to a competition may be detrimental to performance in track and field athletes.

Previous studies reported seemingly contradictory results concerning the impact of cortisol on performance. Cortisol was found to correlate positively with performance in weightlifters^26, 27^ and rugby players^14^ and negatively in tennis players^30^ and golfers^32^. This discrepancy might reflect the different requirements in different sports or differences in study design. Regarding the former, the challenges a weightlifter faces in competition are both physically and mentally very different from those that occur during a golf tournament. Study design might influence the impact of endocrine changes on performance. Differences in study design, for example the importance of the occasion (competition or training) could also influence the impact of hormone levels on performance. In important competitions most athletes might be at or above the optimal level of arousal.

Cortisol, but not testosterone, levels rose between 24 h prior to immediately prior to the competition. The increase in cortisol indicates that a competition at this level is a severe physiological stressor. The fact that testosterone levels did not rise in the last 24 h before the competition is interesting because such an increase is generally expected in anticipation of a challenge in both women^36^ and men^10^, although this effect is not always found^7, 37^. The magnitude of the competition itself (in the case of this study an international multisport event) may also have contributed to our findings because athletes may already have experienced increased level of arousal 24 h prior to the competition. Testosterone levels in the athletes may therefore already have been elevated in anticipation of the competition even 24 h beforehand. This could explain our observation that an increase of testosterone, and to a lesser degree, cortisol levels in the last 24 h before the competition was associated with poorer performance. A less important competition might have resulted in different results from the same cohort.

An increase in testosterone prior to a competition is usually not associated with decreased performance. However, Kivlighan *et al*.^37^ also found a negative correlation between increase in testosterone levels in anticipation of a competition and performance in men and women participating in a rowing ergometer competition. Interestingly, in women this effect was found in novice, but not in varsity rowers, whereas in men the effect was found in both groups. Pregame rise in testosterone has been reported to be related to aggressiveness as well as being focused in female collegiate rugby players^36^. Further, the rise in testosterone before a competition might be associated with a willingness to take risks^38, 39^ and higher testosterone and cortisol levels have been reported to be positively correlated with neuromuscular performance^28^. These aspects are supposed to increase performance, however it can be speculated that taking too much risk or acting too aggressively in a competition might be detrimental to performance. Here further research is needed.

Anticipatory changes in cortisol or testosterone levels did not correlate with the athlete’s status (*SBP*). Nonetheless, the relationship between hormones and status is an interesting subject for future studies. In this study, we found no correlation between testosterone levels after the competition in relation to performance. Multiple studies have reported testosterone levels to increase after positive outcomes in competitions. This includes tennis players^13^ as well as basketball and soccer fans whose team had won^40^ and voters whose preferred candidate won an election^41^. Other studies failed to find an increase in testosterone after a positive outcome^16–18, 42^. One study found an increase in testosterone after a loss in competition to be positively correlated with the participant’s willingness to compete again, whereas no overall difference in post competition testosterone was found between winners and losers^43^. Since the participants in our study were competitive athletes, we assume that an unsatisfactory competition outcome would create a desire to compete again and do better, which can lead to a post competition increase in testosterone. This effect works in the opposite direction of the more frequently described effect that testosterone levels increase after a positive outcome. It is therefore possible that these two effects offset in this study. Quality of sleep may have also had an impact on hormone profiles in the athletes. A recent study demonstrated that athletes frequently do not sleep well before major competitions^44^, and sleep restriction has been reported to lead to altered diurnal cortisol secretion patterns^45^ and reduced testosterone levels^46^. How well an athletes sleeps before a competition could therefore affect his or her hormone levels.

For this study we have not combined data from different competitions, choosing instead to sample a single competition of international importance. This is based on the concern that competitions of varying importance levels might in turn have varying effects on hormonal responses. This might increase variation and would initially outweigh the benefit of increased sample size. We did, however, combine the sexes in our statistical analysis because we found no significant differences in the *∆H* values of males and females and because previous research has shown associations between testosterone and competition outcome to be similar in men and women^47^. Nonetheless, future studies would benefit from increased sample sizes, which would also allow for a separate analysis of male and female athletes and athletes competing in different disciplines. Such a sample size would require international cooperation at high-level competitions.

An intriguing way to validate and extend the results from this study would be to apply the same methodological approach to competitions of differing importance to athletes, as well as to incorporate training conditions. Such studies could reveal how low and high stress conditions (i.e., pressure in relation to event importance) are associated with hormone expression rates and athletic performance. Increased sample size would further enable examining the effects of different settings and conditions during competition, such as the impact of the opponent’s status. Further, increased sample size would enable studies on whether the relationship between status, hormones and performance differs between athletes from different disciplines.

We conclude that the tournament represented a significant stressor for the athletes and that those with a less pronounced endocrine response in the 24 h prior to the competition fared better than those exhibiting greater endocrine changes.

## Electronic supplementary material

Supplementary Information
