# Supplementary material for: Status, Stress and Performance in Track and Field Athletes during the European Games in Baku (Azerbaijan)
Source: Sci Rep. 2017 Jul 20;7:6076. doi: 10.1038/s41598-017-06461-z (PMC5519747; doi:10.1038/s41598-017-06461-z)
Supplement: Supplementary file 1 — Supplementary Information [file 41598_2017_6461_MOESM1_ESM.pdf]

Status, Stress and Performance in Track and Field Athletes during the European Games in Baku (Azerbaijan)

Benjamin Siart[1,2*], Alfred Nimmerichter[3], Claudia Vidotto[4], and Bernard Wallner[2]

1) Department of Anthropology, University of Vienna, Althanstrasse 14, Vienna, Austria

2) Department of Behavioural Biology, University of Vienna, Althanstrasse 14, Vienna, Austria

3) Faculty of Training and Sports Sciences, University of Applied Sciences Wiener Neustadt, Johannes Gutenbergstrasse 3 2700 Wiener Neustadt

4) Study Lab G.m.b.H., Davidgasse 87-89, 1100 Vienna, Austria

*Corresponding author;

Althanstraße 14

A- 1090 Vienna

Tel. +43-680-33255-38

Tel. +43-1-4277-54716

Email: benjamin.siart@univie.ac.at

Keywords: Stress; status; sport; cortisol; testosterone; competition

Supplementary Tables

Supplementary Table 1. Mean cortisol values and standard deviation (sd) and standard error (se) for all sample points

Time	N	Cortisol (ng/ml)	sd	se
pre-comp 24 h	19	3.89	1.77	0.41
m-comp	19	6.07	2.96	0.68
pre-comp	18	7.57	3.72	0.88
post-comp	14	9.21	4.84	1.29
post-comp 1 h	14	6.64	3.42	0.91
post-comp 3 h	15	3.52	1.48	0.38
m post-comp	17	5.41	1.32	0.32

24 h pre-comp = 24h before the Call Room. M-comp = morning of competition. Pre-comp = before entering the Call Room. Post-comp = immediately after competition. Post-comp 1 h = 1 h after competition. Post-comp 3 h = 3 h after competition. M post-comp = morning after competition

Supplementary Table 2. Mean testosterone values and standard deviation (sd) and standard error (se) for all sample points.

Time	N	Testosterone (ng/ml)	sd	se
pre-comp 24 h	16	0.84	0.67	0.17
m-comp	18	0.46	0.62	0.15
pre-comp	16	1.33	1.46	0.36
m post-comp	15	0.47	0.37	0.10

24 h pre-comp = 24 h before the Call Room. M-comp = morning of competition. Pre-comp = before entering the Call Room. M post-comp = morning after competition

Supplementary Figures

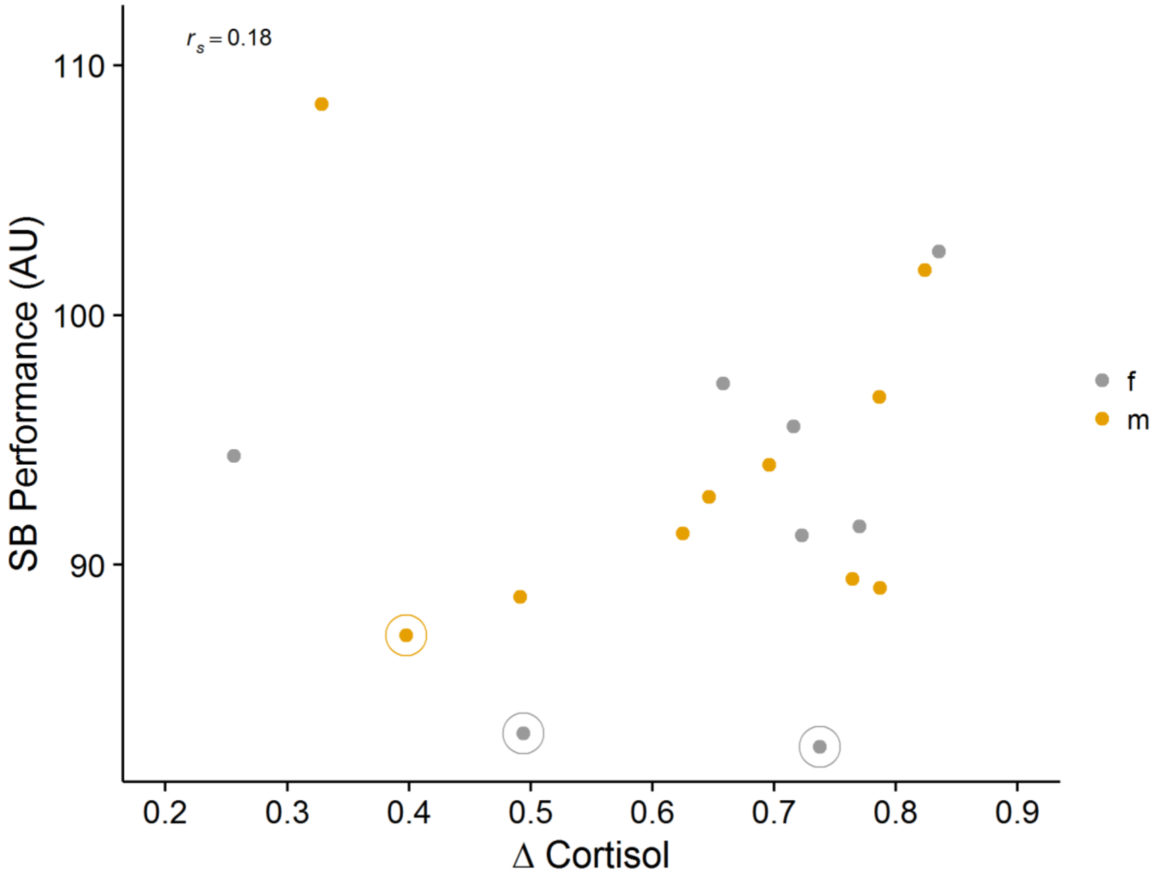

Supplementary Figure 1. Change in cortisol level before competition *ΔC* (X-axis) in relation to the athlete's status in the form of the athlete's season's best in arbitrary units (AU) derived from IAAF scoring points SB (Y-axis). Each symbol represents an individual athlete; color indicates sex of the athlete. Athletes marked with a circle were less involved in competitive athletics than the other athletes.

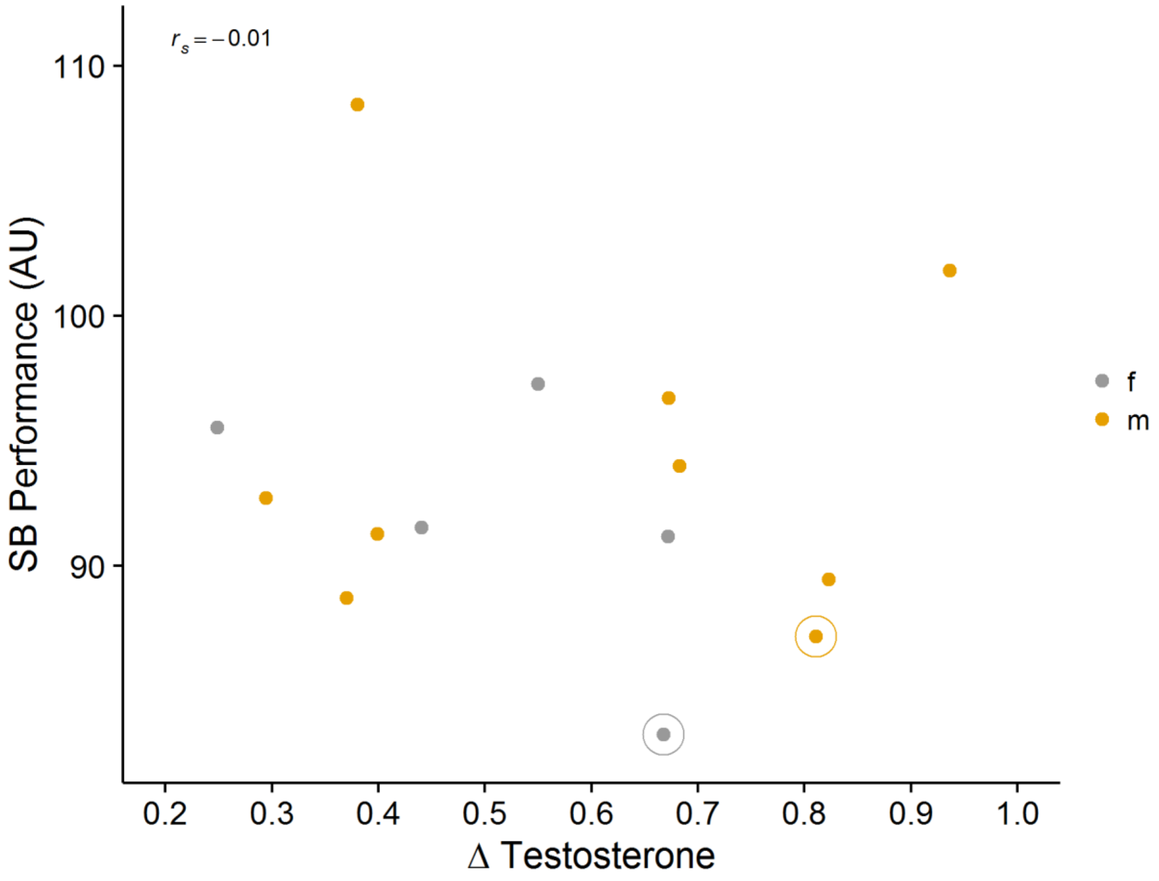

Supplementary Figure 2. Change in testosterone level before competition ΔT (X-axis) in relation to the athlete's status in the form of the athlete's season's best in arbitrary units (AU) derived from IAAF scoring points SB (Y-axis). Each symbol represents an individual athlete; colour indicates sex of the athlete. Athletes marked with a circle were less involved in competitive athletics than the other athletes.

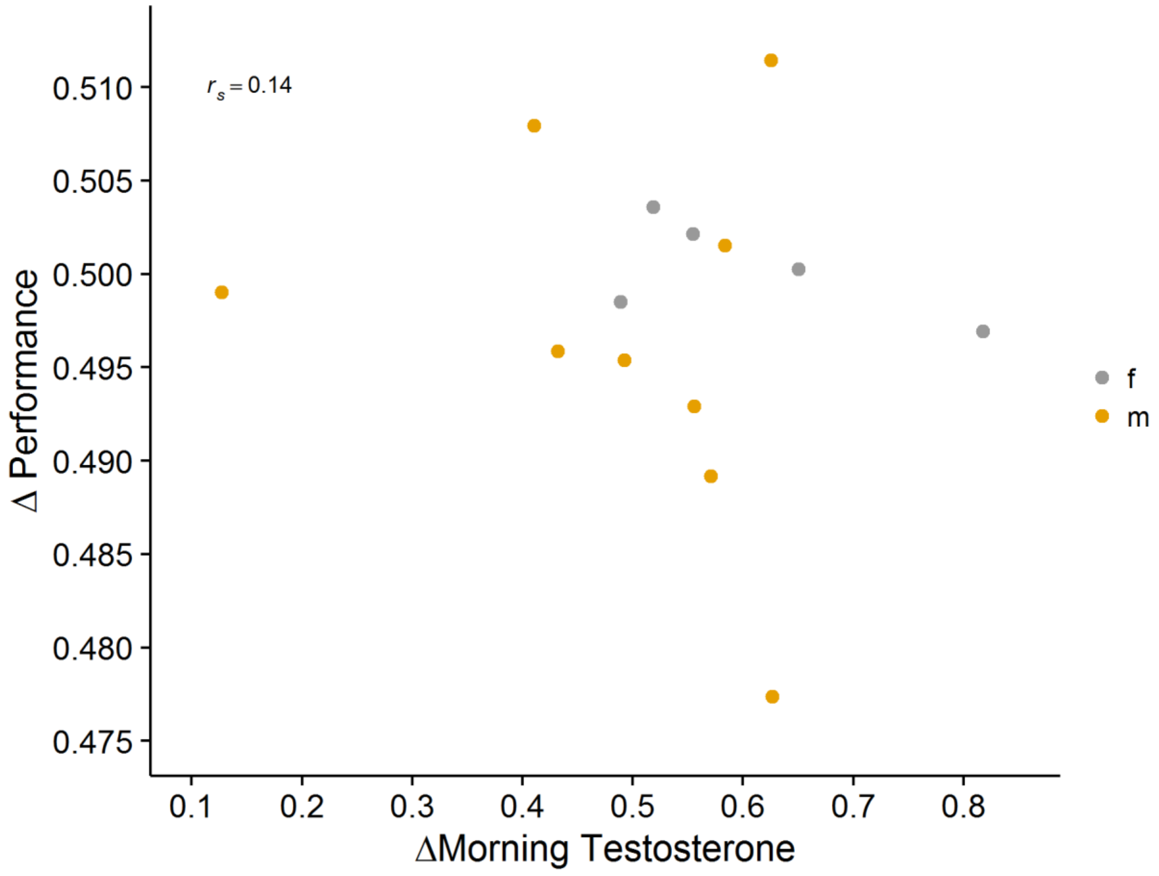

Supplementary Figure 3. Correlation of testosterone change after the competition, $\Delta T_{Morning}$ (X-axis) and performance; ΔP (Y-axis). Each symbol represents an individual athlete; color indicates sex of the athlete.
